# Certificats médicaux pour coups et blessures volontaires en pratique médico-judiciaire à Dakar

**DOI:** 10.11604/pamj.2019.33.225.9291

**Published:** 2019-07-18

**Authors:** Mohamed Maniboliot Soumah, Mor Ndiaye, Yawo Apélété Agbobli, El Hadj Omar Ndoye, Abibatou Dia Sall, Mamadou Lamine Sow

**Affiliations:** 1 Service de Médecine Légale et Médecine du Travail, Faculté de Médecine, de Pharmacie et d'Odontologie, Université Cheikh Anta Diop, Dakar, Sénégal

**Keywords:** Constatations, agressions, consultation médico-judiciaire, Constatations, assaults, medico-judicial consultation

## Abstract

La rédaction du certificat médical est une étape cruciale dans la procédure judiciaire. Il est souhaitable que ce document à visée judiciaire soit rédigé dans une consultation médico-judiciaire. Les objectifs de notre étude étaient d'évaluer la qualité des certificats médicaux dans une consultation médico-judiciaire et d'étudier les critères de validité des certificats médicaux interprétatifs. Il s'agit d'une étude rétrospective portant sur les patients reçus à la consultation médico-judiciaire de l'Hôpital Général de Grand-Yoff, victimes de coups et blessures volontaires du mois d'avril 2012 au mois de février 2015. L'ensemble des certificats médicaux a été inclus dans une fiche d'enquête analysée par Epi Info version 6.04. L'ensemble des 249 certificats étudiés était imprimé en « Arial police 12 », lisible et compréhensible. Tous les certificats médicaux étudiés comportaient une identification complète du médecin et de la victime. La date et l'heure de l'agression figuraient sur 248 certificats (99,6%). Le type de violence subie était précisé sur l'ensemble des 249 certificats avec 164 rixes (65,9%), 64 agressions unilatérales (25,7%). La description précise des lésions figurait sur les 246 certificats où la nature des lésions a été donnée. La localisation des lésions était établie par rapport à des repères anatomiques fixes, dans les différents plans de l'espace. La consultation médico-judiciaire, coordonnée par le médecin légiste, a permis d'améliorer sensiblement la qualité des documents délivrés. Il demeure que la vulgarisation de ces pratiques de rédaction et de délivrance est nécessaire notamment au niveau des hôpitaux régionaux dans le cadre de la formation continue ou post doctorale.

## Abstract (en)

Drawing up the medical certificate is a crucial step in the judicial process. This judicial document should be drafted during a medical legal consultation. This study aims to evaluate the quality of medical certificates in a medico-judicial consultation and to study interpretative criteria of medical certificate validity. We conducted a retrospective study of patient's victims of intentional wounds and blows receiving a medico-judicial consultation at the Grand Yoff General Hospital, from April 2012 to February 2015. The whole of medical certificates were recorded on a survey form analyzed by Epi Info Version 6.04. The whole of the 249 certificates studied was printed in legible and understandable “Arial police 12”. All the studied medical certificates included complete identification of the physician and the victim. The date and the time of aggression were written in 248 certificates (99.6%). The type of abuse suffered was specified on the whole of the 249 certificates: 164 brawls (65.9%), 64 unilateral aggressions (25.7%). The precise description and nature of the lesions was written in 246 certificates. The site of the lesions was established based on fixed anatomical landmarks in the various body plans. Medico-judicial consultation, coordinated by the forensic physician, helped to significantly improve the quality of the documents issued. The dissemination of these drafting practices and of the practices concerning the issuing of certificates is essential in particular in the regional hospital as part of an ongoing training or postgraduate training.

## Introduction

Les coups et blessures visent essentiellement les actes qui portent atteinte à l'intégrité physique d'un être humain. Cependant, certaines violences psychologiques sont également prises en compte. Ces actes sont dits volontaires lorsque leur auteur a eu la volonté de commettre un acte violent. Ces agressions peuvent survenir dans différents contextes. Les agressions peuvent revêtir plusieurs aspects. Après ces faits toujours traumatisants, un dépôt de plainte dans un service de police ou de gendarmerie constitue dans la plupart des cas une démarche difficile à effectuer, notamment lorsqu'il s'agit de mineurs. La rédaction du certificat médical est une étape cruciale dans la procédure judiciaire. C'est un document qui atteste des examens cliniques d'un médecin conformément aux constatations médicales qu'il est en mesure de faire. Il est souhaitable que ce document à visée judiciaire soit rédigé dans une consultation spécialisée: la consultation médico-judiciaire. Les objectifs de notre étude étaient d'évaluer la qualité des certificats médicaux dans une consultation médico-judiciaire, d'étudier les critères de validité des certificats médicaux interprétatifs, d'identifier les difficultés relatives aux bonnes pratiques dans la délivrance des certificats médicaux des coups et blessures volontaires.

## Méthodes

Il s'agit d'une étude rétrospective portant sur les patients reçus à la consultation médico-judiciaire de l'Hôpital Général de Grand-Yoff, victimes de coups et blessures volontaires munis d'une réquisition ou venant d'eux-mêmes du mois d'avril 2012 au mois de février 2015. Nous avons inclus dans notre étude, toutes les victimes de coups et blessures volontaires reçues dans notre consultation médico-judiciaire, avec établissement d'un certificat médical fixant l'incapacité totale de travail. Nous avons exclu les victimes de coups et blessures involontaires, les agressions sexuelles et les sévices sur mineurs. Pour chaque certificat, les paramètres recherchés étaient le support de rédaction, la lisibilité, l'identité du praticien et de la victime, l'âge de la victime, le sexe, la profession, l'adresse de la victime, le lieu de l'agression, la répartition dans l'espace des agressions, l'heure des agressions, l'agent vulnérant, le délai de consultation, le côté dominant de la victime, la description des blessures psychiques, les lésions physiques retrouvées, la topographie des lésions, le bilan paraclinique (morphologique et/ou biologique) effectué, la prise en charge thérapeutique, le caractère récent de l'agression, la compatibilité des lésions avec l'agression subie et la durée de l'incapacité totale de travail. Les données recueillies ont été saisies sur Epidata et analysées sur Epi Info 6.04. Nous avons calculé les proportions pour les variables qualitatives et les moyennes pour les variables quantitatives. Les graphiques ont été réalisés après exportation des données sur pages.

## Résultats

Au total nous avons dénombré 249 certificats descriptifs de coups et blessures volontaires allant de la période d'avril 2012 à février 2015, et qui ont fait l'objet de notre étude. Les résultats sont rapportés ci-dessous.

**Support:** sur les 249 certificats médicaux étudiés tous étaient rédigés sur papier à entête où figurait le nom de la structure hospitalière et le service.

**Lisibilité et compréhension:** l'ensemble des 249 certificats étudiés était imprimé en « Arial police 12 », lisible et compréhensible.

**Identité du médecin:** tous les certificats médicaux étudiés comportaient une identification complète du médecin avec nom et prénom(s), qualité et adresse (professionnelle).

**Figure 1:**
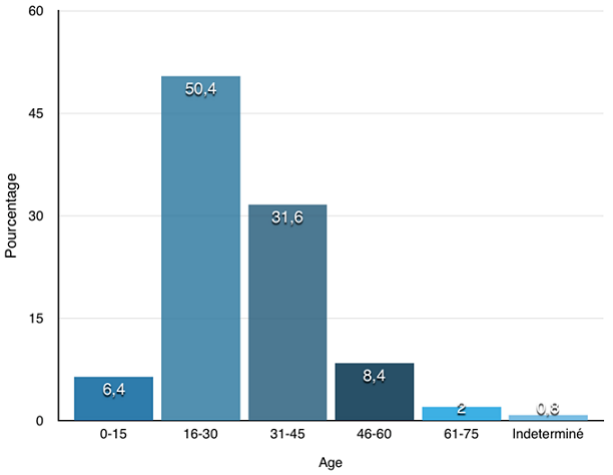
répartition des victimes selon l'âge

**Identification de la victime:** pour l'identification de la victime, nous avons considéré cinq (05) paramètres: nom, prénom(s) et sexe, l'âge ou date de naissance, le côté dominant, l'adresse et la profession: nom, prénom(s) et sexe: le nom, le(s) prénom(s) ainsi que le sexe figuraient sur la totalité des 249 certificats étudiés. Nous comptions 167 hommes (67,1%) et 82 femmes (32,9%) soit un sexe ratio de 2; Age ou la date de naissance de la victime: l'âge ou la date de naissance figurait sur 247 certificats (99,2%). Sur ces derniers, (Figure 1) 126 victimes étaient âgées de 16 à 30 ans (50,4%) et 79 victimes avaient entre 31 et 45 ans (31,6%); côté dominant: le côté dominant figurait sur 242 certificats (97,2%), 226 victimes étaient des droitiers (90,8%) et 16 victimes étaient des gauchers (6,4%); adresse des victimes: l'adresse des victimes figurait sur l'ensemble des 249 certificats étudiés (100%). Selon le découpage adopté pour notre l'étude, les victimes habitaient au centre ville avec 4 cas (1,6%), en zone périurbaine avec 117 cas (47%), à Grand-Yoff et quartier environnant avec 37 cas (14,9%), en Banlieue avec 86 cas (34,5%) et dans une autre région du Sénégal avec 5 cas (2%); profession: l'exercice d'une profession (ou l'absence d'emploi) figurait sur 239 certificats (96%) dont 18 victimes qui étaient sans emploi (7,2%) et 25 victimes qui étaient des élèves et étudiants (10%). Selon les 9 grands groupes de la classification internationale des types de professions (CITP 08) les personnels des services directs aux particuliers, commerçants et vendeurs étaient les plus représentés (28,8%) suivis par les métiers qualifiés de l'industrie et de l'artisanat.

**Figure 2:**
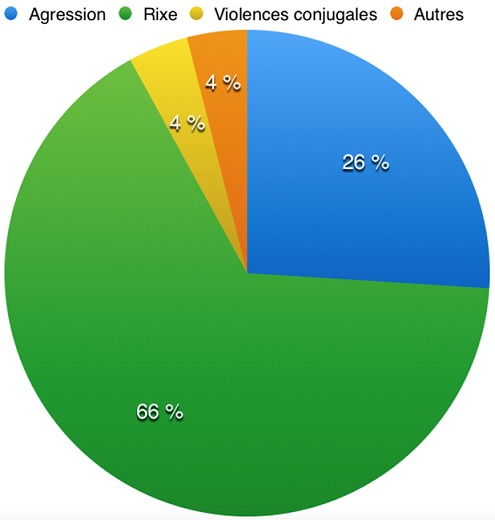
répartition des victimes selon le type de violence

**Commémoratif des faits par le patient:** la date et l'heure d'examen figuraient sur tous les certificats. La date et l'heure de l'agression figuraient sur 248 certificats (99,6%). Nous avions 85 agressions survenues entre 19h-23h (34,1%), 58 agressions entre 13h-18h (23,3%), 56 agressions entre 7h-12h (22,5%) et 49 agressions entre 00h-6h (20,1%). Le lieu de l'agression figurait sur 247 certificats (99,2%) avec 145 agressions survenues sur la voie publique (58%), 82 cas au domicile de l'agresseur ou de l'agressé (32,8%) et 19 cas sur le lieu de travail (8%) et un cas d'agression survenue au sein d'un commissariat de police. Le type de violence subie (Figure 2) était précisé sur l'ensemble des 249 certificats avec 164 rixes (65,9%), 64 agressions unilatérales (25,7%), 11 violences conjugales (4,4%) et 10 cas pour tous autres types de violences (4%). Le type d'arme utilisé figurait sur 244 certificats (98%) avec 104 cas où l'arme utilisée était une arme naturelle (surtout coup de pied, coup de poing,…) soit 42% des cas. Nous avions 64 cas d'utilisation d'armes contondantes (gourdin et projectiles telluriques). Les armes blanches tout type confondu (plus souvent le couteau ou tesson de verre) étaient retrouvées dans 61 cas (24,4%). Nous notions 15 cas où le type d'arme était mixte c'est-à-dire que l'agresseur ou les agresseurs auraient utilisé aux moins deux types d'armes différentes (6%). Le nombre d'agresseur figurait sur 231 certificats (92,8%). Nous notions 179 victimes avec un seul agresseur (72%), 16 victimes avec 2 agresseurs (6,4%), 15 victimes avec 3 agresseurs (6%), 13 victimes avec 4 agresseurs (5,2%) et enfin 8 victimes pour lesquelles le nombre d'agresseurs était supérieur à 4 (3,2%).

**Délai de consultation:** le délai de consultation figurait sur 248 certificats (99,6%) dont 228 victimes qui avaient consulté dans les 10 jours suivant l'agression (dont 17 victimes qui avaient consulté le jour même de l'agression) soit 91,6%, 13 victimes avaient consulté entre le 11^ème^ et le 20^ème^ jour (5,2%), 4 victimes entre le 21^ème^ et le 30^ème^ jour (1,6%) et 3 victimes (1,2%) au-delà du 30^ème^ jour.

**Existence d'antécédents:** la précision sur l'existence ou non d'antécédent figurait sur 237 certificats (95,2%) avec 7 cas où il y avait des antécédents n'interférant pas avec les lésions traumatiques (2,8%) et 230 cas sans antécédents retrouvés (92,4%).

**Figure 3:**
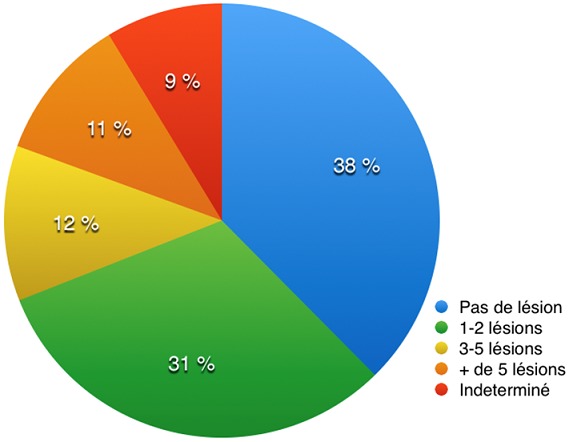
répartition des victimes selon le nombre de lésions

**Examen:** sur les 249 certificats étudiés l'évaluation psychologique a été faite (100%) avec 21 victimes présentant un choc psychologique (8,4%). Nous trouvions 147 cas où le nombre de lésions (Figure 3) était compris entre 1 à 2 lésions (59%) et 76 victimes présentant entre 3 à 5 lésions (30,6%) et 20 victimes présentant plus de 5 lésions (8%). Les lésions étaient le plus souvent mixtes pour 138 victimes (55,4%). Lorsqu'elles étaient uniques, nous comptions 56 plaies (22,4%), 28 contusions (11,2%), 10 fractures (4%), 5 luxations (2%), 4 érosions (1,6%), 3 entorses (1,2%) et 2 morsures (0,8%). La description précise des lésions figurait sur les 246 certificats où la nature des lésions a été donnée et était absente sur 3 certificats correspondant aux certificats où aucune lésion n'a été trouvée. Ces lésions étaient décrites dans leur aspect, leur forme, leur taille précise et les lésions en forme notamment les morsures étaient soulignées dans la description initiale. Sur 227 cas les lésions étaient récentes (91,2%) et 19 victimes présentant des lésions anciennes (7,6%). La localisation des lésions figurait sur 244 certificats (98%) avec 55 lésions (22,1%) localisées au niveau du massif crânio-facial et du cou, 6 lésions du thorax (2,4%), 2 lésions de l'abdomen, 10 lésions du rachis et du bassin (4%), 68 lésions des membres (27,3%) dont (54 cas soit 21,7% pour les membres supérieurs et 14 cas soit 5,6% des cas pour les membres inférieurs) et 103 cas où la localisation des lésions était mixte (41,4%). Cette localisation était établie par rapport à des repères anatomiques fixes, dans les différents plans de l'espace.

**Examen para-clinique:** sur l'ensemble des 249 certificats étudiés, 141 victimes ont effectué des examens para-cliniques (56,6%) avec 125 radiographies (50,2%) toutes topographies confondues, 6 scanners (2,4%), 2 cas pour tout autre type d'examens para-cliniques (0,8%) et enfin 8 cas où au moins 2 types d'examens para-cliniques ont été demandés (3,2%).

**Traitement:** dans notre étude, 245 victimes (98,6%) avaient bénéficié d'un traitement dont 120 par traitement fonctionnel (48,2%), 69 sutures (27,7%), 33 soins locaux (13,3%), 19 traitements orthopédiques (7,6%) et enfin 4 traitements chirurgicaux (1,6%).

**Figure 4:**
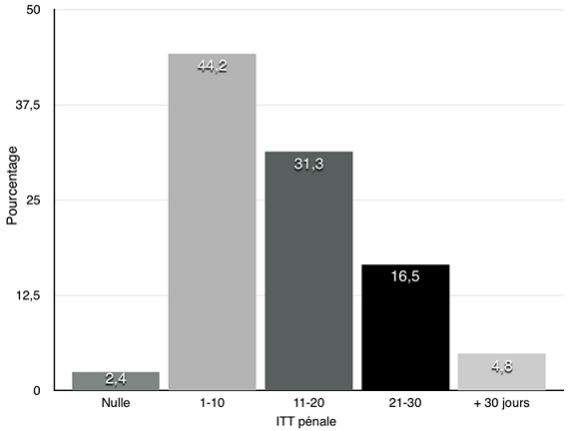
répartition des victimes selon l'ITT pénale

**Conclusion:** l'imputabilité a été appréciée dans l'ensemble des certificats avec 231 cas où il y avait une compatibilité entre les lésions décrites et le type de violence allégué (92,8%) et 18 cas (7,2%) où il y avait une incompatibilité entre les faits allégués et les lésions retrouvées. La compatibilité avec des lésions de défense était soulignée dans la conclusion. L’Incapacité totale de travail (l’I.T.T) Pénale (Figure 4) était présente sur 247 certificats (98,2%) avec 6 cas où l'I.T.T Pénale était nulle (2,4%), 110 certificats avec une I.T.T Pénale entre 1 et 10 jours (44,2%) et 78 certificats avec une I.T.T Pénale entre 11 et 20 jours (31,3%). Le seuil pénal est de 21 jours au Sénégal. L'ensemble des 249 certificats étudiés était signé et l'expression « remis en mains propres » y était mentionnée. En résumé, nous avions des certificats médicaux de bonne qualité remplissant les critères de validité indispensables et les critères relatifs (Tableau 1).

**Tableau 1:** répartition des critères de validité indispensables et de validité relatifs des certificats médicaux

Des critères de validité indispensables							
Paramètres	Identité médecin	Identité patient	Circonstances de rédaction	Commémoratif	Examen clinique	Conclusion	Signé et remis en mains propres
Résultats (%)	100	96	100	100	100	98,2	100
**Critères de validité relatifs**							
**Paramètres**	**Support**	**Lisibilité**	**Délai consultation**	**Antécédents**	**Para-clinique**	**Traitement**	
Résultats (%)	100	100	99,6	95	100	100	

## Discussion

La date exacte et l'heure d'examen figuraient sur la totalité des certificats (100% des cas). Ce paramètre trouve son intérêt dans le fait qu'il puisse permettre d'établir une compatibilité ou non entre la date des faits allégués par la victime et la lésion retrouvée. En effet, certaines lésions comme les ecchymoses ont des caractères colorimétriques qui sont fonction de l'âge de la lésion et selon Grill *et al*. [1] la mention de la date et l'heure de l'examen corrélée à la date et l'heure d'agression peut permettre d'établir cette imputabilité. Le commémoratif des faits allégués par le patient est indispensable. Ce sont des allégations qu'il est préférable de retranscrire au conditionnel ou de les mettre entre guillemets en veillant à ne pas les dénaturer [2]. Ceci permettra au médecin, après examen du patient, de dire en conclusion si les faits allégués par la victime sont en adéquation avec ses constatations. Dans notre étude, le commémoratif des faits figurait sur la quasi-totalité de nos certificats. Les lésions physiques retrouvées figuraient sur 98,8% des certificats et étaient de nature différente, il y avait 3 certificats où aucune lésion physique n'a été trouvée. Dans notre étude, nous trouvions une prédominance des plaies (22,4%) chez les victimes présentant un seul type de lésion. D'autres études réalisées en France [1, 2] et au Maroc [3, 4] ont individualisé d'autres lésions prédominantes. En effet, pour Benyaich *et al*. ce sont les hématomes et les ecchymoses qui prédominaient (51%) suivies des plaies en seconde position, résultat similaire à celui de Louarn *et al*. [2] qui dans leur étude ont également trouvé une prédominance des hématomes et ecchymoses. Pour l'étude de Grill *et al*. l'équipe de Toulouse, notait une nette prédominance des ecchymoses, des dermabrasions et des brûlures [1]; les plaies ne venaient qu'en 4^ème^ position. Cependant dans notre étude, nous notions que certains patients présentaient des lésions mixtes et représentaient la presque moitié de notre étude (48,6%). La mission du rédacteur doit comporter un examen clique soigneux, une description clinique et une discussion médico-légale [5, 6].

La rédaction du certificat médical est une étape cruciale dans la procédure judiciaire. La volonté des autorités médicales et judiciaires sénégalaises, manifestée lors de plusieurs rencontres notamment en 2009, de disposer d'informations fiables en matière de coups et blessures, nous avait permis de mettre en place une consultation médico-judiciaire à Dakar. Cette étude fait suite à des études antérieures faites par notre équipe [7-9] qui avaient ressorti trois problèmes: la mauvaise qualité des certificats médicaux délivrés, la méconnaissance des médecins de la notion d'incapacité totale de travail au sens pénal et la «course aux certificats de 21 jours». Nos résultats montrent, l'amélioration significative dans la rédaction des certificats médicaux par rapport aux études antérieures selon les critères comme suit: la date, la lisibilité et la signature du praticien hospitalier; la mention de l'heure de réception de la victime, de sa profession, ainsi que son côté dominant; le lieu et l'heure de l'agression; le type d'agression et d'armes utilisées; le nombre d'agresseurs; la présence de description des blessures psychiques et physiques de la victime; la précision des examens complémentaires et des traitements administrés; la correcte évaluation de l'ITT pénale.

Dakar capitale du Sénégal, concentre le 1/3 de la population sur moins de 0,28% du territoire national. Nous exerçons dans la seule consultation spécialisée en matière de violence. C'est pourquoi, nous recommandons: la création d'unités médico-judiciaires, notamment dans les différentes régions du Sénégal, permettant une meilleure collaboration entre médecins et juges. Ces structures formeront un complexe qui permettra aux victimes de bénéficier d'une prise en charge multidisciplinaire; la confection d'un guide de rédaction des certificats médicaux de coups et blessures volontaires disponible au niveau de toutes les structures sanitaires afin d'aider les médecins à évaluer l'ITT pénale chez les victimes de coups et blessures; la formation des médecins, des avocats, des officiers de police judiciaire, des juges à la notion d'ITT pénale pour leur permettre de distinguer l'ITT pénale de l'« arrêt de travail ».

## Conclusion

La rédaction du certificat médical est essentielle en pratique médicolégale du vivant. Cela participe à la sanction pénale et en définitive au maintien de l'ordre public. Cette pratique a connu une avancée au Sénégal avec la mise en place d'une consultation médico-judiciaire à Dakar, à l'Hôpital Général de Grand-Yoff. Outre la sécurisation du processus de délivrance de ces certificats, limitant la délivrance de certificats de complaisance, cette consultation médico-judiciaire, coordonnée par le médecin légiste, a permis d'améliorer sensiblement la qualité des documents délivrés. Pour tous les critères étudiés les scores atteints sont supérieurs à 96%. Il demeure que la vulgarisation de ces pratiques de rédaction et de délivrance est nécessaire notamment au niveau des hôpitaux régionaux dans le cadre de la formation continue ou post doctorale.

### État des connaissances actuelles sur le sujet

- Il y a peu de données relatives au sujet car dans l'espace Afrique noire francophone, il y a deux consultations médico-judiciaires, l'une à Conakry, l'autre à Dakar objet de notre étude;
- Les médecins connaissent peu la notion d'incapacité totale de travail au sens pénal;
- Les certificats médicaux de coups et blessures délivrés par les médecins sont en général mal libellés et incomplets comme nous le démontrions dans nos études antérieures.

### Contribution de notre étude à la connaissance

- Au vu de nos résultats antérieurs, nous avons mis en place une consultation spécialisée après nos travaux de 2011, la première du genre au Sénégal, dont nous évaluons l'activité;
- Les certificats délivrés par les spécialistes du dommage corporel sont de meilleure qualité avec une maitrise des différents concepts dont l'imputabilité et la notion d'incapacité totale du travail au sens pénal;
- Cette consultation pilote doit être vulgarisée et développée dans toutes les structures hospitalières car elle sécurise et améliore la délivrance des certificats médicaux de coups et blessures.

## Conflits des intérêts

Les auteurs ne déclarent aucun conflit d'intérêts.
